# Movement patterns during gait initiation in older adults with various stages of frailty: a biomechanical analysis

**DOI:** 10.1186/s11556-024-00335-w

**Published:** 2024-01-13

**Authors:** Jana Maria Hommen, João P. Batista, L. Cornelius Bollheimer, Frank Hildebrand, Thea Laurentius, Hannah Lena Siebers

**Affiliations:** 1 Department of Cardiology, St. Vinzenz-Hospital, Cologne, Germany; 2 Department of Geriatric Medicine, Uniklinik RWTH Aachen, Aachen, Germany; 3 Chair for Physiotherapy, SRH University of Health, Leverkusen, Germany; 4 Department of Orthopedic, Trauma and Reconstructive Surgery, Uniklinik RWTH Aachen, Aachen, Germany

**Keywords:** Biomechanical gait analysis, Gait initiation, Fried frailty phenotype, Fall prevention, Eldercare strategies, (three-dimensional) motion capture system

## Abstract

**Background:**

Gait initiation is challenging for older individuals with poor physical function, particularly for those with frailty. Frailty is a geriatric syndrome associated with increased risk of illness, falls, and functional decline. This study examines whether spatial and temporal parameters of gait initiation differ between groups of older adults with different levels of frailty, and whether fear of falling, and balance ability are correlated with the height of lifting the food during gait initiation.

**Methods:**

Sixty-one individuals aged > 65 years, classified by Fried frailty phenotype, performed five self-paced gait initiation trials. Data was collected using a three-dimensional passive optical motion capture system, consisting of 10 cameras with the ability to perceive reflective markers, and two force plates. The total duration of gait initiation and the duration of its four sub-phases, the first step length, and the maximum foot clearance during the first step were derived, and compared statistically between groups. Additionally, an association analysis was conducted between foot clearance and fear of falling, and confidence in balance in older individuals.

**Results:**

Frail individuals had significantly longer unloading durations, and total durations of gait initiation compared to non-frail older adults. Additionally, they had shorter first step lengths compared to non-frail older adults. Pre-frail older adults also showed shorter steps compared to the non-frail group. However, there were no significant differences between groups for the maximum foot clearance during the first step. Nevertheless, the maximum foot clearance of older individuals correlated significantly with their fear of falling and confidence in balance.

**Conclusion:**

Older adults with reduced physical function and signs of frailty mainly display longer duration of gait initiation and decreased first step length compared to non-frail older adults. The release phase is decreased as the double support phase is prolonged in frail patients. This information can guide the development of specialized exercise programs to improve mobility in this challenging motion between static and dynamic balance.

## Introduction

The population of older adults is increasing rapidly, both in numbers and as a share of the total. According to the World Population Prospects report published in 2022, the share of the global population at ages 65 and above is projected to rise from 10% in 2022 to 16% in 2050 [1]. This demographic change necessarily requires novel eldercare strategies that can efficiently cope with the growing burden of healthcare and medical costs [2]. One particular challenge is the healthcare of aging people with frailty. Frailty is characterized by decreased physiologic reserves and resistance, increased vulnerability to acute stressors, and an overall decline in functional capacity [3–5]. In most cases, it is related to aging, disability, and comorbidity - however, because of its complexity and multidimensionality, it is a difficult term to conceptualize [6]. Fried et al. [4] first developed a phenotype of frailty, which defines it as a clinical syndrome considering various symptoms and signs with a distinct focus on musculoskeletal function. Currently, this is the most frequently used approach for identifying affected patients in clinical practice and research settings [5, 7]. The Fried phenotype includes five criteria to determine if a person is frail or not: Exhaustion, self-reported unintentional weight loss, low physical activity, as well as objective measures of weak grip strength and slow gait speed. Individuals with three or more present criteria are considered as „frail“, those with one or two criteria as „pre-frail“, and those with no criteria of the above as „non-frail“. As frail older adults are particularly prone to falls, report particularly frequently on fear of falling, and frequently suffer from postural stability disorders and gait abnormalities [8–10], a detailed assessment of selected biomechanical parameters of gait could provide new insights as to how interventions might be designed to improve ambulatory capabilities in this vulnerable population.

Gait initiation (GI) is the transient phase from a quiet standing posture to steady state walking [11–15]. It requires the integration of different sensory information from the somatosensory, vestibular, and visual systems, as well as the coordination of multiple skeletal muscles [16, 17]. Deficits in these functional areas lead to an increased potential risk for falls [18–20]. Since most falls in older persons occur due to the inability to respond appropriately to an impaired balance and its ineffective compensation [21], parameters during GI may be sensitive indicators for detecting previously hidden issues and diseases.

**Fig. 1:**
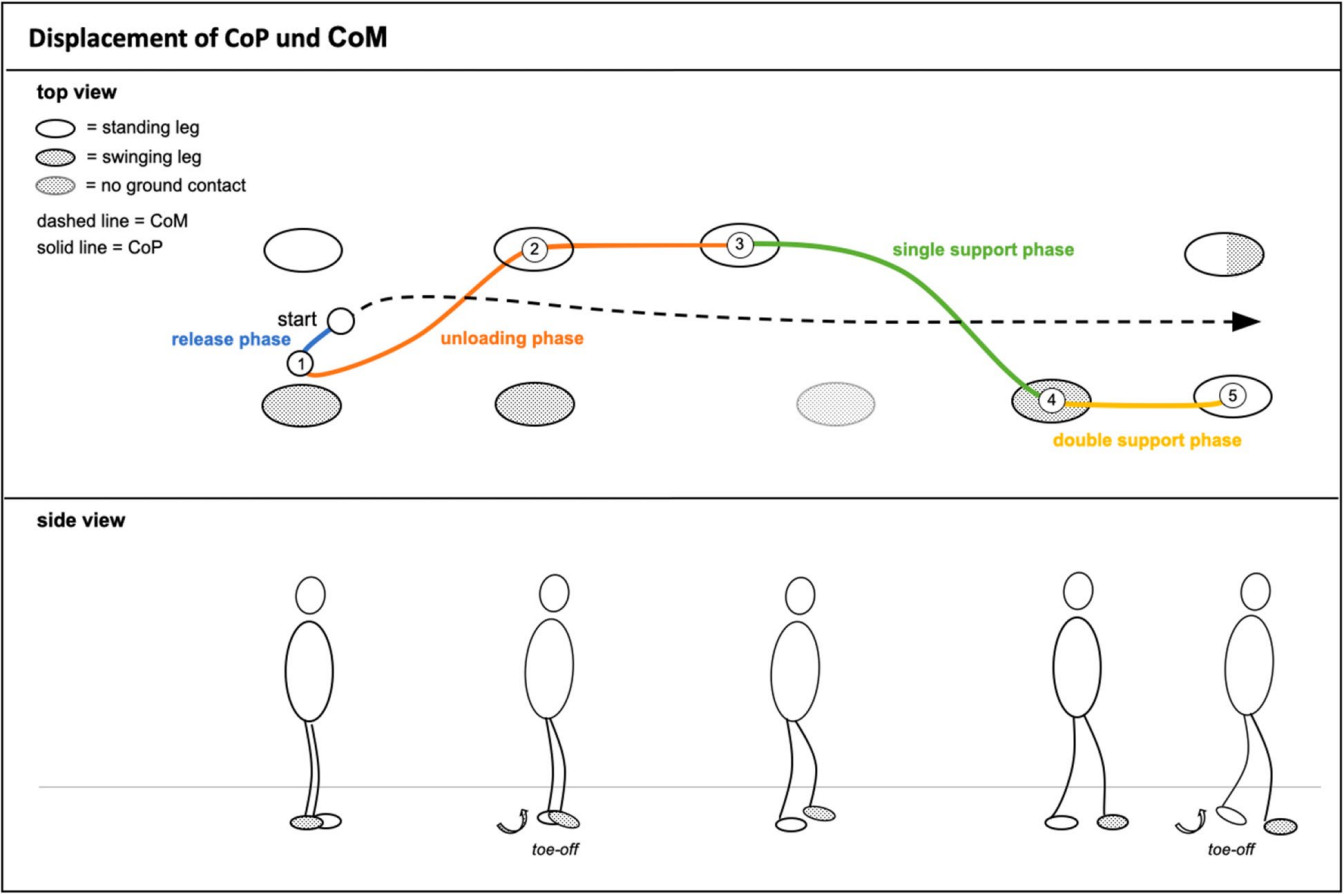
Phases of gait initiation *Notes*. Overview of the phases of gait initiation and the characteristic pattern of center of pressure (CoP, colored solid line), and center of mass (CoM, dashed black line) displacement during each sub-phase

GI can be described by two main phases: As shown in Fig. 1, after the preparatory (postural) phase (also called “anticipatory postural adjustments”), follows the phase of actual stepping (“execution phase”) [22–24]. During the preparatory phase, the center of mass (CoM) decouples from the center of pressure (CoP), thereby giving the body the necessary momentum to fall forward about the ankle joint [25–27]. This phase can be divided into two sub-phases: a release phase and an unloading phase [28]. During the release phase, the CoP is shifted toward the swing leg, resulting in an increasing horizontal ground reaction force thereby accelerating the CoM in the opposite direction [29]. It lasts until the farthest posterolateral movement of the CoP (① in Fig. 1), and its change in direction marks the beginning of the following unloading phase. Here, the CoP moves rapidly toward the stance leg (② in Fig. 1), thus unloading the swing leg for step execution (③ in Fig. 1) [27]. The second main phase, the execution phase, starts as soon as the swing leg is no longer in contact with the ground and ends with the toe-off of the initial stance leg. It can be subdivided into a single support phase and a double support phase. The single support phase lasts from the toe-off of the swing leg until it contacts the ground again (④ in Fig. 1), leading to the double support phase which ends with the toe-off of the prior stance leg ⑤ in Fig. 1 [27].

Previous studies that have utilized GI to evaluate postural control have been mostly limited to age-related changes without considering the interindividual differences regarding health status and functioning [9]. In addition, previous studies have mainly focused on biomechanical parameters during the preparatory phase, omitting parameters during the actual execution of the very first step. However, impairments of measurable parameters during step execution have been shown to correlate with fall events [30–32]. Therefore, examining all sub-phases of GI in frail older individuals is crucial for a deeper understanding of the effects of frailty on motor patterns in this fall-prone population. This analysis could also provide the basis for improved diagnostics and targeted therapies, ultimately reducing the incidence of falls and improving the overall quality of life for this vulnerable population.

### Aim

This study examines whether spatial and temporal parameters of gait initiation differ between groups of older adults with different levels of frailty, and whether fear of falling, and balance ability are correlated with the height of lifting the food during gait initiation. We hypothesize that, due to poor physical function, the different sub-phases, and, consequently, the total duration of GI is elongated, and the first step length is shortened in older adults with frailty compared to age-mates without frailty. Regarding the maximum foot clearance during the first step, two opposite assumptions are conceivable: Either, also because of poor physical condition, the foot is raised lower or, because of increased fear of falling and lower confidence in balance, it is higher as a protective mechanism.

## Methods

### Participants

All participants were grouped based on the score of Fried’s Frailty phenotype model [4] into the groups “Non-frail” (*n* = 36, frailty score = 0), “Pre-frail” (*n* = 14, frailty score = 1 or 2), and “Frail” (*n* = 11, frailty score = 3, 4 or 5) (see Table 1 for details).

**Table 1:** Means ± Standard Deviations of characteristics of the study participants and the GI variable per group and results of ANOVA on differences between the means

Criteria	Non-frail (*n* = 36)	Pre-frail (*n* = 14)	Frail (*n* = 11)	F(2,58)	*p*	Effect size (η^2^)
**Age (years)**	72.89 ± 6.02	75.07 ± 4.73	78.91 ± 7.62	4.22	**0.019**	0.13
**Height (cm)**	168.56 ± 7.37	166.86 ± 6.67	166.55 ± 9.58	0.43	0.653	0.02
**Weight (kg)**	73.55 ± 14.14	76.38 ± 16.93	74.66 ± 20.94	0.16	0.856	0.01
**BMI (kg/m**^**2**^**)**	25.71 ± 3.56	27.31 ± 5.04	26.76 ± 6.24	0.73	0.488	0.02
**Test scores**						
**FES-I**	17.81 ± 1.72	19.31 ± 2.84	22.91 ± 7.71	7.90*	**< 0.001**	0.22
**ABC**	95.44 ± 5.84	86.79 ± 10.21	68.70 ± 22.87	22.69	**< 0.001**	0.44
**GI variables**						
**Release phase duration (s)**	0.25 ± 0.04	0.23 ± 0.03	0.24 ± 0.05	1.32	0.274	0.04
**Unloading phase duration (s)**	0.28 ± 0.05	0.31 ± 0.09	0.34 ± 0.06	4.38	**0.017**	0.13
**Single support phase duration (s)**	0.40 ± 0.04	0.40 ± 0.05	0.43 ± 0.05	2.73	0.074	0.09
**Double support phase duration (s)**	0.16 ± 0.05	0.18 ± 0.05	0.21 ± 0.05	4.85	**0.011**	0.14
**Total duration (s)**	1.08 ± 0.11	1.12 ± 0.15	1.22 ± 0.13	6.05	**0.004**	0.17
**Step length step 1 (m)**	0.58 ± 0.08	0.50 ± 0.09	0.44 ± 0.07	14.13	**< 0.001**	0.33
**Max. FC during the first step (cm)**	4.30 ± 1.26	3.79 ± 0.10	4.16 ± 1.29	0.92	0.403	0.03

Note. *p*-values < 0.05 are indicated in bolt. For FES, the F-value is given for 2 and 57 degrees of freedom. According to Cohen [33], the limits for the size of the effect are 0.01 (small effect), 0.06 (medium effect) and 0.14 (large effect)

Included in the study were participants able to walk without walking aids. Exclusion criteria were: cognitive impairment (< 24 points in the Mini-Mental-Status-Test (MMSE) according to Folstein et al. [34] or a severely limited mobility that precludes independent care (e.g., bedridden). The latter was determined during a screening interview and was considered fulfilled if the participant was largely able to move independently within the home. Participants with severe visual impairments, uncontrolled cardiovascular disorders, uncontrolled Parkinson’s syndrome, acute chronic obstructive bronchitis, or acute states of confusion (e.g., delirium) were also not eligible to participate in this study.

All participants gave their written and oral consent. The study was approved by the independent medical Ethics Committee at the RWTH Aachen Faculty of Medicine (ethics committee number 142/18).

### Instruments

The study was performed in the motion analysis laboratory of the department of geriatric medicine of the university hospital RWTH Aachen, Germany. A three-dimensional optical motion capture system (Qualisys AB, 5+ series, Göteburg, Sweden) with 10 cameras tracked the marker trajectories at 120 Hz. In total, 52 reflective markers were placed at anatomical landmarks on participants’ bodies following a prescribed marker set protocol [35]. The calibrated anatomical system technique (CAST) was used to place and determine the movement of segments. The measurements were done using Qualisys Track Manager (Version 19.1, Qualisys AB, Gothenburg, Sweden). After markers labeling at the Qualisys Track Manager software, raw data were exported to .c3d for further analysis with the software Visual 3D (Version 6.0, C-Motion. Inc., Germantown, MD, USA). Force data were recorded by two force plates (Bertec Corporation, Columbus, Ohio, USA), which were embedded in the surface in the middle of a 10-m walkway. The movement and force data were filtered using a fourth-order low-pass Butterworth filter with a cut-off frequency of 5 Hz.

### Frailty assessment

In all participants, the five criteria of Fried’s phenotype of frailty [4] were assessed before GI data collection: unintentional weight loss, subjectively perceived fatigue, low physical activity, slow walking speed, and muscle weakness. For this purpose, questions were first asked about unintentional weight loss of more than 5 kg within the last year and about subjectively perceived fatigue. Last-mentioned was done by the “Fatigue assessment according to Fried”, which takes up two questions of the Center for Epidemiologic Studies Depression Scale [36]. Using a short version of the Minnesota Leisure Time Physical Activity Questionnaire [37], physical activity was assessed by asking about various leisure time activities within the last 4 weeks. Walking speed was measured over a 4.57 m walking distance, and finally, to detect possible muscle weakness, strength measurement of the dominant hand was performed three times with calculation of the mean value. We used Fried’s cut-off values to assess the grip strength. One point was awarded for each deficit in one of the five categories. If one to two categories are fulfilled, the classification as “pre-frail” is made, from three as “frail”. Moreover, to evaluate the fear of falling and the balance ability of the old participants, the questionnaires of the Falls Efficacy Scale-International (FES-I) [38, 39] and the Activities-Specific Balance Confidence Scale (ABC) [40, 41] were collected before starting the GI trials.

### Experimental protocol to analyze gait initiation

For the measurement process, each participant was initially asked to stand quietly on a force platform in a relaxed posture on both legs. Both feet were then placed in a parallel position on the first force plate with the toes close to the second one. The width was not dictated and should correspond to their natural stance. Acquisition of force and motion data was triggered, just before the participants received a verbal cue, to begin walking. In response to the cue, they initiated gait with their leading leg at their usual walking pace until the end of the movement lab which corresponded to a walking distance of about 4 m. To become familiar with the experimental protocol, each participant first performed a practice trial. The practice trial was then immediately followed by five data collection trials. Each participant had the opportunity to take a break after a trial to avoid any exhaustion effects. For the study, every participant wore comfortable clothing, including a t-shirt, shorts, and anti-slip socks.

### Calculation of the biomechanical parameters

To describe GI, it was subdivided into the four sub-phases described above. Parameters, including the duration of the release phase (s), unloading phase (s), single support phase (s), and double support phase (s), were calculated. We used specific events to automatically identify the distinct phases by using the analysis software Visual 3D. The start of GI, and therefore also of the release phase, was defined to be 0.15 s before the minimum velocity of the CoP in the walking direction. The furthest point of posterolateral CoP displacement then marked the beginning of the unloading phase. The following single support phase started as soon as the toes of the swinging leg lost contact with the ground. The last sub-phase, the double support phase, was defined by the recontact of the heel of the swinging leg with the ground and ended with the lift-off of the toes of the initial stance leg.

The total duration of GI and the percentages of each sub-phase on the total duration of a respective study participant were calculated separately.

The length of the first step (m) was calculated between the first toe off-event of the swing phase and the initial contact of the foot with the force plate.

The maximum foot clearance (max. FC) during the first step (m) was calculated by the maximum value of displacement of a marker at the midfoot. The marker is a virtually created marker, whose position was determined centrally, i.e. at a 50% distance between the real markers at the toe and heel of the feet.

### Statistical analysis

Descriptive statistics (mean and standard deviation (SD)) were calculated for demographic data (age, height, weight, and BMI) and each determined parameter from the arithmetic mean values of the five trials per person. Between-group differences in the total duration of GI, the durations of the four sub-phases, the step length of the first step, and the max. FC were tested with a one-way analysis of variance (ANOVA) for continuous variables. Bonferroni-adjusted post-hoc analysis was used during follow-up testing.

Since the duration of the sub-phases is also affected by a change in the total duration of the GI, we found that the relative durations of the sub-phases to the total duration of the GI is another interesting aspect to illuminate. Therefore, we additionally calculated the relative proportion (%) of the phase durations of the sub-phases in relation to the total duration of the GI. Therefore, group differences in the percentages of the different phase durations of GI were tested statistically with a one-way multivariate analysis of variance (MANOVA). Follow-up tests on separate univariate ANOVAs were conducted when appropriate. The level of significance was 0.05.

In addition to comparing the max. FC during the first step between groups, we also investigated the results of the FES-I and ABC for a possible correlation with this parameter. This was done for all participants (*n* = 61) to determine if there is an correlation between max. FC and fear of falling and/or confidence in balance, regardless of the participant’s frailty score. For this purpose, Spearman’s rank correlation was computed for each case. The interpretation of the effect strength was based on the classification according to Cohen [33]. Accordingly, the effect limits are 0.10–0.29 (weak), 0.30–0.49 (moderate), and greater or equal to 0.5 (strong).

All statistical tests were performed using IBM SPSS Statistics Version 27 (Armonk, New York, USA).

## Results

Of initially 92 adults measured, 31 participants were not included in the data analysis due to insufficient data acquisition and/or quality and the application of exclusion criteria. Ultimately, the data of *n* = 61 participants aged 65 years and older were analyzed. The female to male ratio was 20/16, 8/6, and 2/9 for the non-frail, pre-frail and frail group, respectively. There were no significant differences between the groups with respect to height, weight, and BMI, but age and the scores FES-I and ABC (Table 1). Pre-frail people were not significantly older than the non-frail participants, but the mean age of the frail group was 6 years higher than that of the non-frail group (about 79 compared to 73 years) which was significant (Table 2). The frail group showed a significantly higher FES-index than the non-frail group, and a significantly increased ABC index than both, non-frail and pre-frail group.

**Table 2:** Results of the post-hoc tests for variables with significant differences between groups

Dependent variable	Groups compared	Mean difference	95% Confidence Interval		*p*
			Lower Bound	Upper Bound	
**Age**	Non-frail - pre-frail	−2.18	−6.90	2.54	0.776
	Non-frail - frail	−6.02	−11.18	−0.86	**0.017**
	Prefrail - frail	−3.84	−2.20	9.87	0.367
**FES**	Non-frail - pre-frail	−1.50	−4.48	1.48	0.657
	Non-frail - frail	−5.10	−8.28	−1.93	**< .001**
	Prefrail - frail	−3.60	−7.38	0.17	0.066
**ABC**	Non-frail - pre-frail	8.66	− 0.34	17.65	0.063
	Non-frail - frail	26.74	16.90	36.58	**< .001**
	Prefrail - frail	18.08	−29.59	−6.58	**< .001**
**Unloading phase duration (s)**	Non-frail - pre-frail	−0.03	−0.08	0.02	0.440
	Non-frail - frail	−0.07	− 0.12	− 0.01	**0.018**
	Prefrail - frail	−0.03	−0.10	0.03	0.601
**Double support phase duration (s)**	Non-frail - pre-frail	−0.02	−0.06	0.02	0.452
	Non-frail - frail	−0.05	−0.09	− 0.01	**0.011**
	Prefrail - frail	−0.03	−0.08	0.02	0.454
**Total GI duration (s)**	Non-frail - pre-frail	−0.04	−0.14	0.06	0.925
	Non-frail - frail	−0.15	−0.26	− 0.04	**0.003**
	Prefrail - frail	−0.11	−0.24	0.02	0.103
**Step length step 1 (m)**	Non-frail - pre-frail	0.09	0.02	0.15	**0.005**
	Non-frail - frail	0.14	0.07	0.21	**< .001**
	Prefrail - frail	0.05	−0.03	0.13	0.375

Note. *p*-values < 0.05 are indicated in bolt

The GI variables unloading phase duration, double support phase duration, the total duration, and length of step 1 showed significant differences between the three groups (Table 1). For release phase duration, single support phase duration, and maximum FC during the first step, the differences between groups were not significant.

**Fig. 2:**
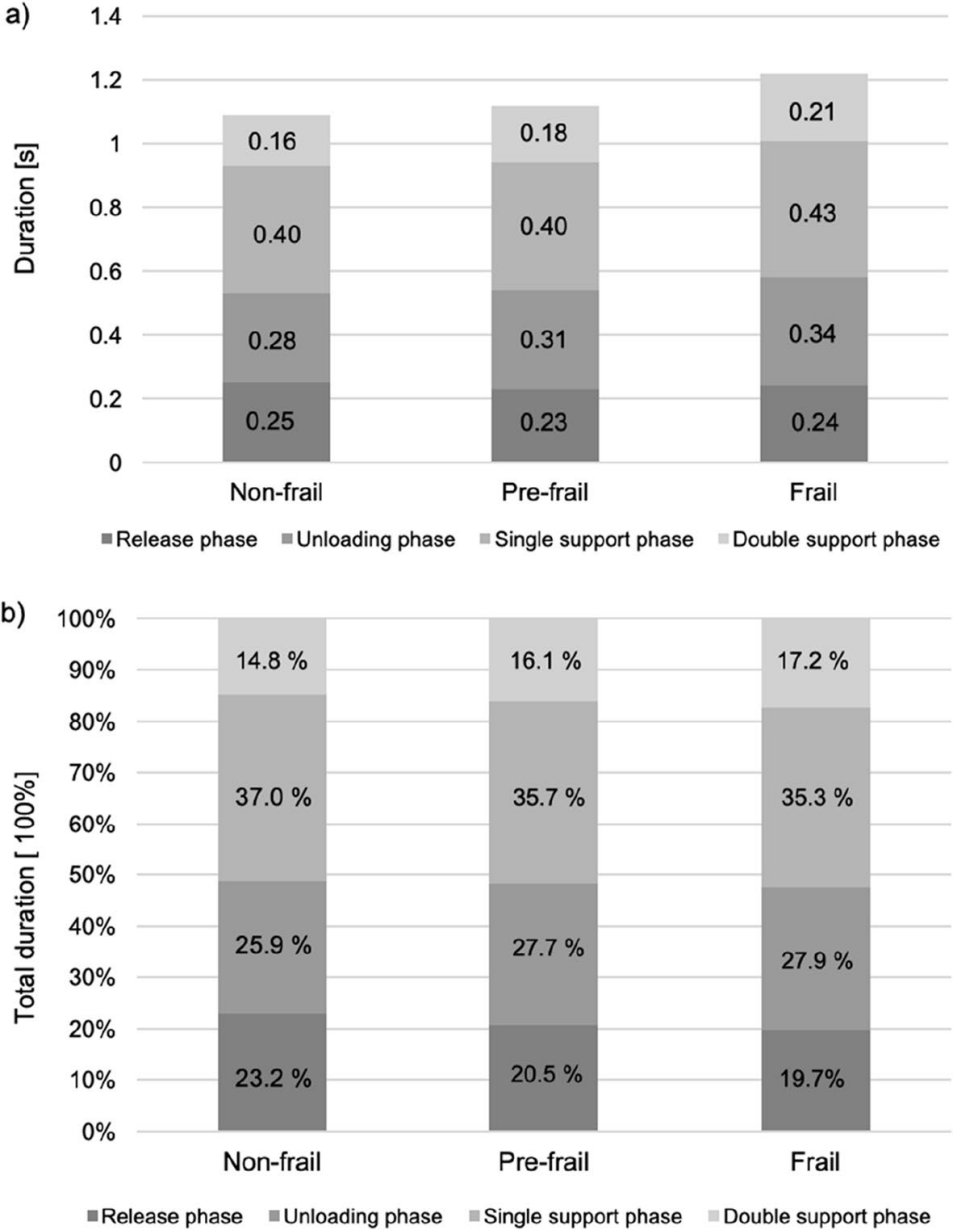
Graphic illustration of the changes in the durations *Notes. *Total duration of GI and its division into the four sub-phases in seconds (**a**), and, in comparison, the distribution of the percentages of the sub-phases (**b**) of total GI duration

Post-hoc tests with Bonferroni corrections revealed that the unloading and double support and, in consequence, total duration of GI was significantly longer in the frail than the non-frail group (Table 2, Fig. 2a). The pre-frail group values were in-between the values of the two other groups without significant differences to both. A one-way MANOVA found no significant differences between the groups regarding the percentages of the sub-phases, *F*(6, 112) = 1.782, *p* = 0.109, partial η^2^ = 0.087). However, on closer inspection of the percentage distribution of the individual sub-phases, it becomes evident that there is a notable increase in the percentage of the unloading phase and double support phase, while there is a corresponding decrease in the percentage of the release phase and single support phase with increasing frailty (Fig. 2b). The length of the first step of pre-frail and frail people was significantly smaller than that of the non-frail group (Table 2).

**Fig. 3:**
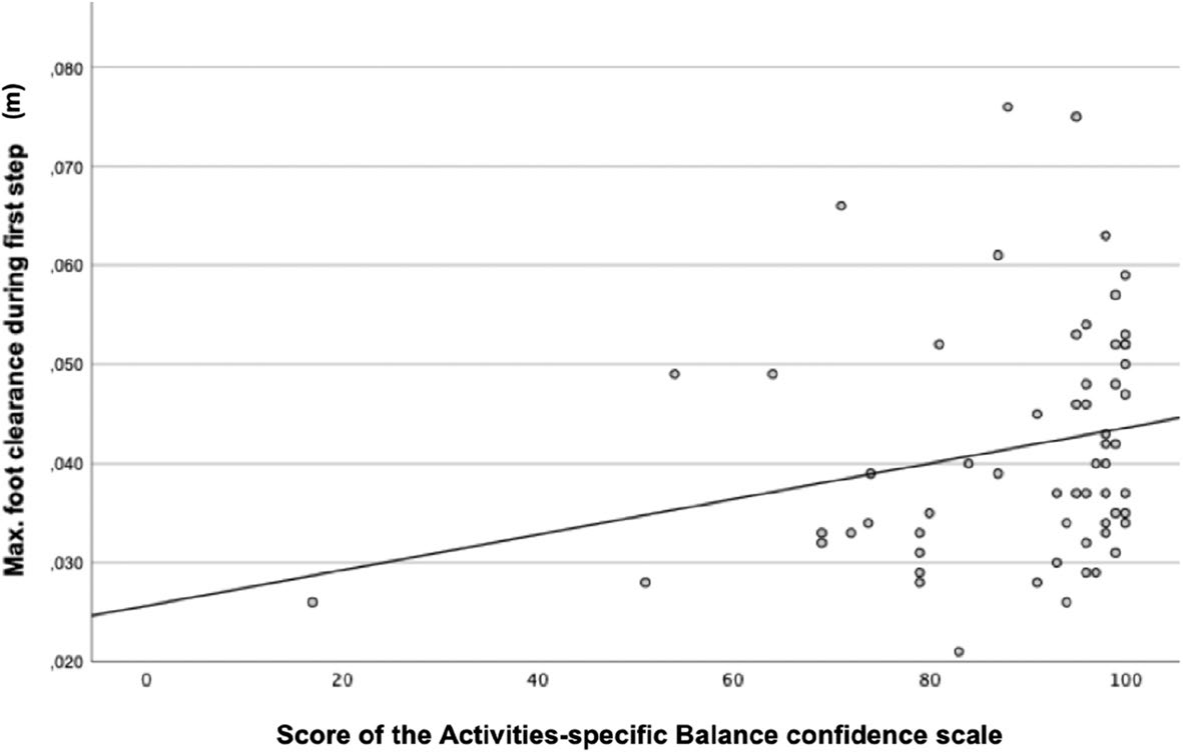
Correlation between the maximum foot clearance and the score of the Falls Efficacy Scale-International questionnaire

**Fig. 4:**
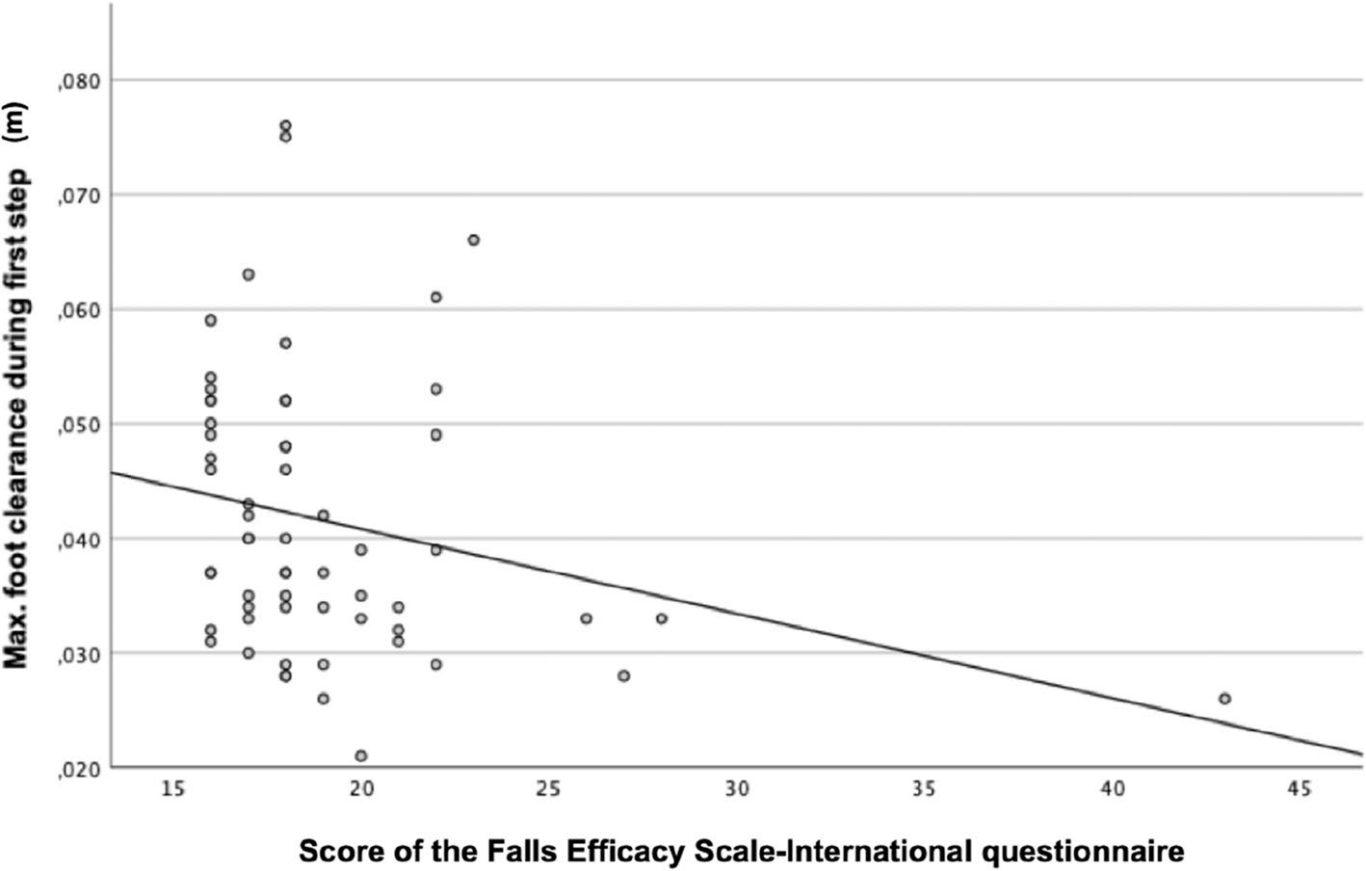
Correlation between the maximum foot clearance and the score of the Activities-specific Balance confidence scale

Spearman’s rank correlation was computed to assess the relationship between the max. FC during the first step and both questionnaires (FES-I and ABC) (see Fig. 3 and Fig. 4). Between the FES-I and the max. FC a moderate negative correlation was found, *r*(59) = − 0.31, *p* = 0.016. Between the ABC and max. FC a moderate positive correlation was found, *r*(59) = 0.32, *p* = 0.012. The higher the fear of falling, or the lower the confidence in balance in old people, the lower the foot was lifted during the first step.

## Discussion

The main goal of this study was to describe and analyze the gait initiation task in old individuals during different frailty stages. Our hypotheses of increased duration for GI and a decreased first step length because of reduced physical function in frail people were supported by our results, whereas for the time no significant difference between pre-frail and frail patients was shown. Apart from the frailty status, older adults show increased duration for GI and shorter step length compared to the young reference group. Previous studies described a gait strategy involving decreased gait speed and shortened step length to stabilize dynamic balance [42, 43]. Slow gait speed in general is one of the most established parameter for defining individuals as pre-frail or frail [42], and has already been the focus of various previous gait analyses, e.g. [43] or Kressig et al. [9]. Notably, when we look at the results for the individual sub-phases, the absolute duration of the release phase during GI did not differ significantly between the groups and was even similar to the young reference group. However, by analyzing the percentage duration of the release phase on the total duration for GI, a noticeable shortening in pre-frail and frail older adults can be observed. During this phase, the CoP first shifts towards the swing leg thereby allowing a forward momentum. A shortening may cause insufficient weight transfer and movement thus leading to a more unstable GI that is prone to falls. The resulting lengthening of both, the percentage and absolute duration of the unloading phase, implies that the CoP moves more slowly toward the stance leg, which in turn leads to the overall slower gait of frail adults. Upon closer examination of the absolute times, the double support phase shows the most recognizable difference besides the total time, which is even more evident in comparison to the young reference group. Also, the percentage double support phase lengthens with increased frailty, and the percentage single support phase shortens. This in turn results in a decreased step length, as evidenced by the decreased step length associated with a higher frailty score. Among others Mbourou et al. [44] and Kwon et al. [45] examined the duration of the double support phase during the GI task period in older fallers, all noticing a much smaller first step length and a longer duration of the double support phase congruent to our findings for frail participants. Besides the absolute time, also the percentage time of the double support phase on GI increased with frailty. Therefore, the lengthening of the double support period has a direct impact on gait characteristics and may be used in frail adults to stabilize their inefficient gait control.

Taken together, our results suggest that frailty leads to a more cautious and conservative gait strategy. This is immediately evident when starting walking, as individuals with frailty tend to maintain dynamic balance by reducing gait velocity and taking shorter steps to prevent falls. Previous studies have already shown that older individuals walk more slowly and with shorter steps than young people [25, 29, 46]. Our research demonstrates that the frailty syndrome leads to a further deterioration of these parameters during GI. Additionally, we found that frailty is associated with a shortened portion of the release phase, indicating uncertainty in walking off. So, in the future, analysis of the GI or the very first step could be sufficient to identify biomechanical parameters that indicate increased gait insecurity and fall risk, without requiring a comprehensive gait analysis.

Except for the first step length, a significant difference was always demonstrated between non-frail and frail adults, while the pre-frail and frail groups did not differ for any of the parameters.

Nevertheless, there were clearly noticeable trends for the groups, which also allow conclusions to be drawn for older individuals with greater frailty: Frail patients experience significant delays in GI compared to non-frail counterparts, without a clear trend in maximum FC. Individuals with higher fear of falling tend to lower their max. FC, aiming to stay closer to the ground, potentially minimizing fall distance and aiding quick recovery. Additionally, a positive association with the ABC score suggests that lifting the foot requires adequate self-confidence in balance. However, the practicability of using FC as a frailty assessment tool remains uncertain.

Some limitations of this study must be considered. In general, the participants should be more evenly distributed in the groups, and the small group size of pre-frail, respectively frail adults, should be considered. For instance, to improve scatter plot distribution, it would be valuable to include participants with higher FES-I and lower ABC scores. Another limitation worth mentioning is that the initial contact of the foot used to delimit one step may have been constituted with either the medial foot or the heel. Although this variability is characteristic of the gait initiation of frail elderly people and, therefore, the movement pattern of this population, it may have affected the temporal and spatial phases of gait initiation.

Analyzing movement patterns of older adults and, particularly, frail individuals proved to be a challenging task. Nevertheless, we were able to identify significant trends. Notably, significant age differences exist between frailty groups, yet analyzing age independently lacks clinical utility. Future research should verify results’ transferability with larger samples, especially including severely frail participants.

We found a moderate correlation between the fear of falling respectively the confidence in balance and the maximum food clearance during the first step. However, the correlation was driven by a single participant with very low confidence in balance respectively high fear of falling. Thus, final conclusions on these correlations should be based on data of a group with a more equal distribution of the fear of falling respectively confidence in balance.

Recognizing that our results were derived from a controlled laboratory setting, their direct application to real-life scenarios might be limited. Our results are to be tested and verified with simpler measurement methods. Inertial sensors as simpler and cheaper devices may provide a valid and more realizable alternative for measuring e. g. overall GI time in clinical routine [32, 47, 48]. Our results, gathered in a controlled lab setting and focusing on straight walking, may not directly apply to real-life scenarios. Factors like footwear, varied terrains, and changing directions in daily activities influence movement and balance differently. Thus, while our findings mark the initial phase for new diagnostic tools, their direct translation to everyday situations might be limited. Yet, these findings pave the way for developing and adapting diagnostic tools for everyday use.

## Conclusion

In summary, we have found that prolonged times for GI and a shorter first step length are associated with higher frailty. Therefore, spatiotemporal parameters in GI exhibit potential as predictors of functional decline and fall risk associated with frailty. This insight could streamline the classification of frail patients, aiding in timely interventions to prevent physical decline and falls. However, while our study points to the potential of targeted exercise interventions, specifically focusing on improving GI times, randomized controlled trials are necessary to validate their efficacy in fall prevention among the elderly.

## Data Availability

The raw data used to support the conclusion of this article are available from the corresponding authors upon request.

## Abbreviations

ABC: Activities-specific Balance confidence scale
ANOVA: One-way analysis of variance
BMI: Body mass index
CAST: Calibrated anatomical system technique
cm: Centimeter
CoM: Center of mass
CoP: Center of pressure
df: Degrees of freedom
*F*(*v*_1_, *v*_2_): *F*-valu*e* with *v*_1_ and *v*_2_ degrees of freedom
FC: Foot clearance
FES-I: Falls Efficacy Scale-International
GI: Gait initiation
Hz: Hertz
Inc.: Incorporated
MANOVA: One-way multivariate analysis of variance
max.: Maximum
m: Meter
MMSE: Mini-Mental State Examination
n: Total number of individuals
η^2^: Effect size
p: *p*-value
R^2^: Coefficient of determination
s: Second
SD: Standard deviation
USA: United States of America
